# Origin and application of the Lorentz factor in X-ray diffraction

**DOI:** 10.1107/S2053273326005917

**Published:** 2026-07-07

**Authors:** Fabian Gasser, Josef Simbrunner, Guillaume Freychet, Nicola Demitri, Václav Holý, Roland Resel

**Affiliations:** a Institute of Solid State Physics Graz University of Technology Petersgasse 16 Graz 8010 Austria; b Department of Neuroradiology, Vascular and Interventional Radiology Medical University of Graz Auenbruggerplatz 9 Graz 8036 Austria; c Université Grenoble Alpes CEA Leti Grenoble 38000 France; d Elettra Sincrotrone Trieste SCpA SS 14 – km 163, 5 in AREA Science Park Basovizza Trieste 34149 Italy; e Department of Condensed Matter Physics, Faculty of Mathematics and Physics Charles University Ke Karlovu 5 Prague 12116 Czechia; Institute of Crystallography - CNR, Bari, Italy

**Keywords:** intensity corrections, Lorentz factor, integrated intensities, single-crystal diffraction, powder diffraction, grazing-incidence diffraction, small-angle scattering

## Abstract

The Lorentz factor is a fundamental correction factor in quantitative analysis of X-ray diffraction experiments, enabling measured integrated peak intensities to be related to calculated structure factors. In this review, the physical origin of the Lorentz factor, its derivation and practical implementations are presented based on a unified approach, treating the Lorentz factor as the Jacobian relating the experimental measurement coordinates to the reciprocal-space volume element. This approach clarifies the trigonometric and wavelength-dependent contributions to the Lorentz factor and explains the origin of the commonly used ‘angular-velocity’ formulation for rotational measurements. Equations for the Lorentz factor are derived systematically for a broad range of modern X-ray diffraction geometries, including single-crystal and powder diffraction, grazing-incidence diffraction methods, as well as small-angle X-ray scattering. Hereby it is demonstrated that the Lorentz factor sensitively depends not only on the experimental geometry, but also on the type of sample under investigation. In addition, we discuss how the Lorentz correction may be avoided by direct reciprocal-space integration, and the practical limitations of such approaches are pointed out. By providing a unified and comprehensive overview of the Lorentz factor, this review aims to support reliable and consistent intensity evaluation across various modern X-ray diffraction methods.

## Abstract 2

This review treats the role of the Lorentz factor in quantitative analysis of X-ray diffraction data. The origin of the Lorentz factor, its derivation and practical implementations for a variety of modern X-ray diffraction methods are discussed.

## 1. Introduction

Quantitative analysis of X-ray diffraction patterns is the basis of several crystallographic techniques, such as crystal structure elucidation or texture determination. Kinematic scattering theory is the easiest approach to evaluate peak intensities for gaining access to the required structure factors. A number of different correction factors – like the Debye–Waller factor, the polarization factor and others – affect the measured intensities (Schwarzenbach, 1996 ▸), so that a detailed knowledge of their influences is of fundamental importance for accurate data analysis and evaluation (Alexandropoulos *et al.*, 2006 ▸).

Among these important factors, the Lorentz factor stands out as particularly critical. Named after Hendrik Antoon Lorentz, the Lorentz factor first appeared in the appendix of a paper on temperature effects by Debye, based on objections made by Lorentz (Debye, 1913 ▸). The Lorentz factor is a geometrical correction factor that is related to the fact that the appearance of a Bragg peak is always associated with the variation of experimental parameters during the intensity measurements required to obtain the total integrated intensity of a selected Bragg peak. Such variations include, for example, changes in wavelength for a fixed crystal or rotational movements of crystals and/or detectors. As a consequence, the Lorentz factor critically depends on the experimental setup, and the use of an incorrect Lorentz factor can substantially reduce the quality of the obtained diffraction data (Cella *et al.*, 1970 ▸).

The derivation of the Lorentz factor for a specific measurement can be performed by two approaches, both of which are reported in the literature and have been applied since the foundations of X-ray crystallography (Darwin, 1922 ▸; Authier, 2013 ▸). In the first approach, the total intensity of a Bragg reflection is determined by integration of the differential (spectral) intensities under consideration of experimental parameters (Laue, 1926 ▸; Als-Nielsen & McMorrow, 2011 ▸). The second approach is based on a simple geometric model for the intensity measurement of a Bragg peak. Here, the transfer of a reciprocal-lattice point through the Ewald sphere is considered, which is associated with a specific time interval that permits the appearance of a Bragg reflection (Darwin, 1922 ▸; Cox & Shaw, 1930 ▸). Correspondingly, the term ‘angular-velocity factor’ is widely used in the literature (Buerger, 1940 ▸; Lipson *et al.*, 2006 ▸).

Based on these two approaches, the Lorentz factors for different experimental techniques have been determined (Kalman, 1979 ▸; Cella *et al.*, 1970 ▸; Lange, 1995 ▸), including those for the fundamental experimental X-ray diffraction geometries on three-dimensional periodic structures: namely, the Laue technique, the rotating-crystal method and powder diffraction (Zachariasen, 1994 ▸). Furthermore, Lorentz factors for specific wavelength-dependent neutron techniques have been derived (Buras & Gerward, 1975 ▸; Zhang *et al.*, 2023 ▸).

## 2. The origin of the Lorentz factor

Following the basic derivation of X-ray diffraction in the kinematical approximation, the diffracted intensity from a crystallite in the far-field limit is described by (Warren, 1990 ▸)
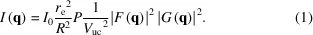
Here, 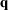 is the scattering vector, 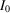 is the intensity of the incident X-ray beam, 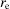 is the classical electron radius, *R* is the distance from the crystal to the detector, *P* is the polarization factor and 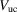 is the volume of the unit cell. Corrections for systematic errors like absorption are neglected for this work (Alexandropoulos *et al.*, 2006 ▸). The structure factor *F* is given by
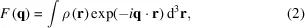
with 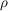 denoting the electron density within the crystallographic unit cell and 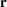 the position vector.

The geometrical factor *G* is defined as the superposition of the Fourier transformations of the crystal shape function *S* at various reciprocal-lattice points:
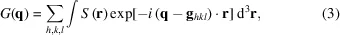
with 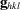 referring to a reciprocal-lattice vector with Laue indices *hkl*. The crystal shape function *S* describes the shape of the crystallite and is defined as unity inside, and zero outside the crystal. Consequently, the introduction of the geometrical factor efficiently separates sample-dependent terms affecting the intensity distribution in a diffraction experiment from other, technique-dependent terms. In the special case of a cuboid-shaped crystal with a defined number of repeating unit cells along the main crystallographic axes *a*, *b* and *c*, the geometrical factor 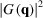 corresponds to a superposition of three slit interference functions (Laue, 1960 ▸; Warren, 1990 ▸).

For most diffraction experiments, the consideration of a continuous intensity distribution in reciprocal space, as described in equation (1), is not practical. Instead, it is more useful to consider the integrated intensities of the individual Bragg reflections appearing in the experiment. This approach is justified when the crystallite is sufficiently large, so that the geometrical factor 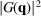 is sharply localized around the reciprocal-lattice vector 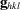 and overlaps between neighboring reflections can be neglected. Under this condition, the structure factor 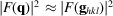 may be regarded as constant across an individual reflection. The integration of equation (1) over a finite reciprocal-space volume 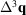 surrounding a single reflection then reduces to an integration over the geometrical factor 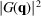, for which in Appendix *A*1[App appa] it is shown that
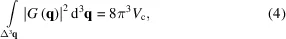
where 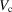 denotes the coherently irradiated volume of the crystallite. Combined with equation (1), the integrated intensity of a reflection with Laue indices *hkl* can therefore be expressed as
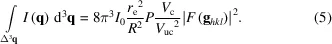
The crystallite volume 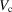 therefore acts as a scattering invariant, an essential property for conventional diffraction experiments, as it permits the use of relative integrated intensities. The integration of a diffraction peak over a finite reciprocal-space volume 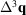 and its relation to the crystallite volume is only applicable if the crystal is sufficiently large so that neighboring reflections do not overlap and there is sufficient space in between reflections to define clear integration limits. For smaller crystals, an alternative, generally applicable formulation is provided in Appendix *A*2[App appa].

Clearly, equation (4) is only valid if the integration over 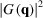 is performed over the volume element in reciprocal-space coordinates 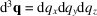. If the peak integration of equation (5) is performed over any coordinates other than reciprocal-space coordinates, the measured data need to be corrected by a Jacobian for the specific coordinate transformation (Laue, 1960 ▸). Although different conventions are found in the literature, we define the inverse of this Jacobian as the Lorentz factor (Kalman, 1979 ▸; Shayduk, 2010 ▸). In other words, the Lorentz factor relates a volume element in the measurement (real-space) system to a volume element in reciprocal space.

Historically, the integration of peak intensities had to be performed on the detector itself, so that the application of a Lorentz factor related to the real-space coordinates of the detector in the specific measurement setup was inevitable. On modern X-ray diffraction setups, the use of large area detectors combined with the possibility to numerically treat large datasets, in principle, allows measured diffraction data to be transformed to reciprocal-space coordinates. In this case, numerical peak integration can be performed directly in reciprocal space following equation (5) without requiring the use of any Lorentz factor (Shayduk, 2010 ▸; Drnec *et al.*, 2014 ▸). Nevertheless, in practice, peak integration in real-space coordinates is still widely applied, as it avoids extensive numerical treatment of large datasets and thereby allows fast data reduction.

In the following sections, the Lorentz factor for different experimental setups will be derived based on the introduced fundamental concept of a Jacobian relating the real-space measurement coordinates to the reciprocal space. Hereby, particular focus will be put on three different X-ray diffraction techniques which are currently widely applied. The rotating-crystal method is the basis of current single-crystal investigations, powder diffraction is used for polycrystalline materials with randomly oriented crystallites, and grazing-incidence X-ray diffraction is the state-of-the-art method for investigating crystalline thin films and surfaces. Finally, a short discussion about the relevance of the Lorentz factor in small-angle X-ray scattering will be given.

## 3. Single-crystal X-ray diffraction

The success of a single-crystal X-ray diffraction experiment for structure elucidation critically depends on the reliability of the integrated intensities of measured Bragg reflections. Most modern single-crystal diffraction setups are built based on the principle of a rotating single crystal. In the following, a detailed derivation of the Lorentz factor for the rotating single-crystal method is given, followed by a short discussion about the Lorentz factor for the historically relevant Laue method on fixed crystals.

### 3.1. Rotating single-crystal method

**Figure 1:**
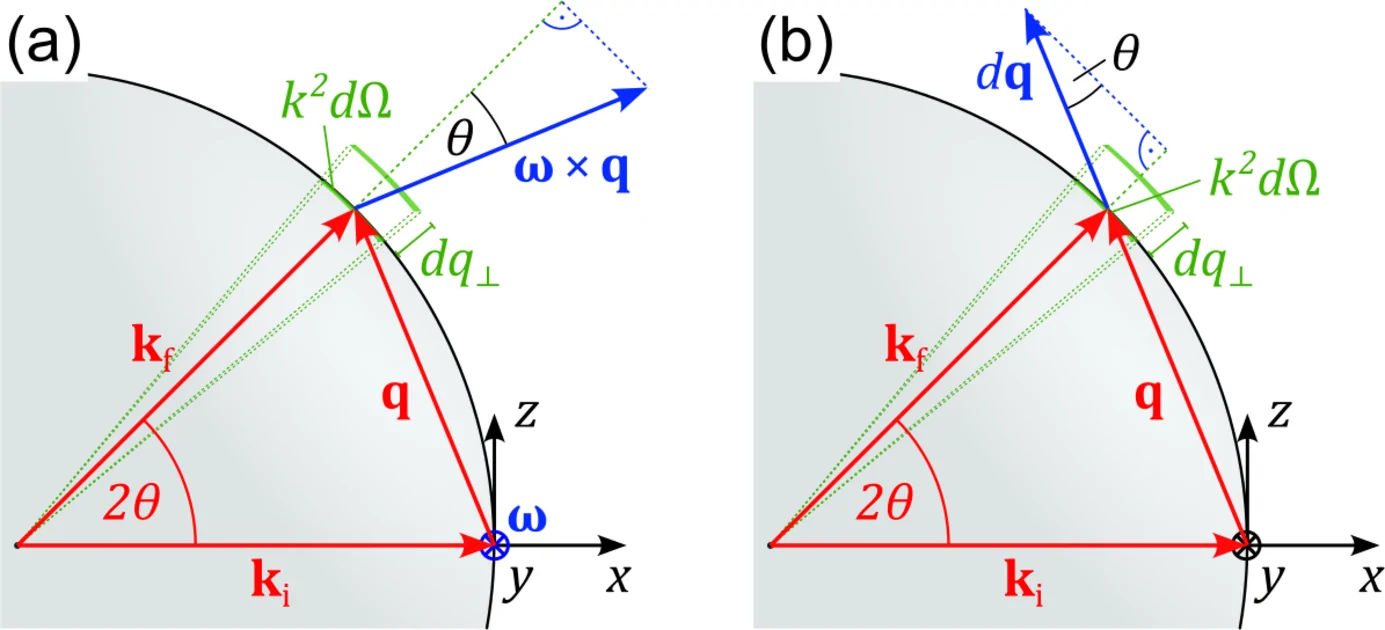
Reciprocal-space representation of different single-crystal X-ray diffraction methods required for the derivation of the respective Lorentz factor. (*a*) For the rotating single crystal, the reciprocal-lattice points are rotated with an angular velocity 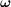 through the fixed Ewald sphere defined by the wavevector of the incident beam 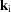, with the detector covering the reciprocal area element 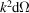 around the wavevector of the diffracted beam 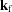. (*b*) In the Laue method, polychromatic radiation is used, so that a change of the scattering vector 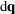 related to a change of the length of the wavevector *k* needs to be taken into account.

The rotating single-crystal method is based on the principle of a fixed detector and a rotating single crystal that is illuminated with monochromatic radiation. The reciprocal-space representation of the setup of a rotating single-crystal experiment is visualized in Fig. 1 ▸(*a*). For simplicity of illustration, the wavevector of the incident beam 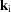 and the wavevector of the diffracted beam 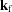 are shown to be in the *xz* plane, and the rotation axis of the single crystal described by angular velocity 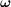 aligns with the *y* axis. The direction of the incident wavevector defines the Ewald sphere and the difference between incident and scattered wavevectors gives the scattering vector 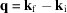. If the detector is kept at a fixed position, it captures the intensity diffracted in a certain solid angle along the surface of the Ewald sphere. This is described by the reciprocal area element 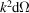, where 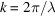 is the length of the wavevectors 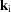 and 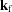, and 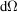 is the corresponding solid-angle element on the Ewald sphere. Consequently, a volume element in reciprocal space can be expressed as
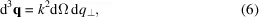
where 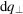 describes a reciprocal length element perpendicular to the Ewald sphere.

For the rotating single-crystal experiment, the Ewald sphere remains fixed throughout the entire experiment, while the reciprocal-lattice points of the crystal are rotated through the Ewald sphere. The reciprocal length element 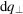 thus has to be expressed in terms of the motion of the reciprocal-lattice points through the Ewald sphere at the position 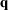:
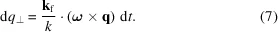
Here, 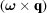 describes the velocity of a reciprocal-lattice point at the position 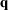. The scalar product with the normalized wavevector 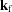 gives the projection of the velocity onto the Ewald sphere normal, ensuring that the obtained 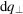 is perpendicular to the Ewald sphere. By inserting this expression into equation (6), the Lorentz factor for a rotating single crystal is obtained as the Jacobian (Milch & Minor, 1974 ▸)
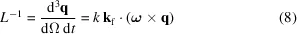
relating the real-space integration variables 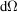 and d*t* to the reciprocal volume element 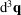.

The introduced derivation conceptually shows why the Lorentz factor is frequently denoted as the ‘angular-velocity factor’. Namely, in the case of the rotating single crystal, the trigonometric part of the Lorentz factor is given by the normal velocity component at which a reciprocal-lattice point moves through the Ewald sphere at the position 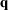. Complementary to this geometrical derivation of the Lorentz factor, Appendix *B*1[App appb] presents a direct algebraic approach, expressing the reciprocal-space vector as a function of the experimental variables and calculating the Jacobian directly.

The Lorentz factor from equation (8) proves to be very convenient for the application to measured data, as it is not only valid for a specific orientation of 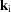, 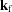 and 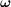, but is generally applicable to any reflections observed in a rotating single-crystal experiment with an arbitrary rotation axis (Milch & Minor, 1974 ▸). The shown equation is also essential for the application of the Lorentz factor to diffraction measurements performed on state-of-the-art area detectors. Hereby each detector pixel acts as a tiny point detector counting photons over time. For a specific measurement setup, together with a freely selectable crystal rotation axis (defined by 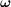), the Lorentz factor can be calculated for each detector pixel and applied directly to the measured data before peak integration.

It is important to remember, however, that the Lorentz factor was derived for a fixed solid-angle element 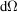. In the case that different detector pixels cover different solid-angle elements, an additional solid-angle correction needs to be applied to the measured data (Gasser, Simbrunner *et al.*, 2025 ▸; Jiang, 2015 ▸). In the simple case of a point detector mounted at a fixed distance *R* from the crystal, the solid-angle element may be expressed as
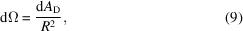
where 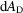 is an area element of the detector.

By further restricting the experimental setup to a coplanar scattering geometry [as shown in Fig. 1 ▸(*a*)], the incident and scattered wavevectors are fixed to be in the same plane perpendicular to the rotation axis, so that 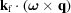 = 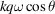 and 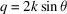, and equation (8) reduces to
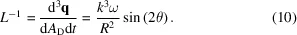
Here, 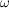 is the angular velocity and 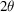 the scattering angle. The obtained equation gives the most common expression for the Lorentz factor reported in the literature (Lipson *et al.*, 2006 ▸; Als-Nielsen & McMorrow, 2011 ▸). Combining the obtained Lorentz factor with equation (5), the widely recognized formula for the integrated intensity of a Bragg reflection is obtained (Warren, 1990 ▸):
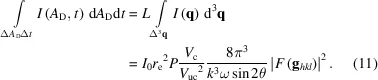


It is important to emphasize, however, that this equation is not generally applicable to an arbitrary diffraction experiment. Instead, it is only valid for a rotating single-crystal experiment in the coplanar scattering geometry, where the detector counts the photons reaching the detector within a fixed area over time. In particular, this implies that the factors appearing in the Lorentz factor (*i.e.* the wavevector *k* and the scattering angle 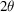) are constants during the measurement of a specific reflection. They only become critical when comparing the intensities of different reflections measured at different scattering angles, or potentially at different wavelengths.

An alternative approach for measuring the integrated intensity of Bragg reflections of a single crystal is the rocking-curve measurement. Here, the crystal remains fixed while the incident and scattered wavevectors scan across a reciprocal-lattice point maintaining a fixed scattering angle 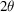. In reciprocal space, both rocking-curve measurement and the rotating single-crystal method perform the identical relative movement through the Ewald sphere. Correspondingly, the Lorentz factor for both methods is equally defined through equation (10). Rocking-curve measurements are only rarely applied; however, measuring the mosaic spread of a reflection can provide a useful estimate of the quality of a single crystal (Davis & Stempel, 1921 ▸; Masiello *et al.*, 2014 ▸).

### 3.2. Laue method

While the rotating single-crystal and the rocking-curve techniques use monochromatic radiation, the Laue method utilizes polychromatic white radiation. Correspondingly, in the Laue method, the diffraction pattern of a single crystal can be measured statically without the need for any crystal or detector rotations. The simplicity of a measurement based on the Laue method is, however, connected to high complexity in the data evaluation. For that reason, the method is only rarely applied, although it was essential for the development of X-ray crystallography (Authier, 2013 ▸).

As the Laue method utilizes polychromatic X-rays, the intensity of the primary beam 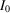 in equation (1) needs to be expressed as a primary spectral intensity 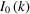. Correspondingly, the method cannot be represented in reciprocal space by a single Ewald sphere, but by a superposition of Ewald spheres corresponding to the different wavevectors *k*. Nevertheless, it is useful to consider a single Ewald sphere with radius *k* as depicted in Fig. 1 ▸(*b*), where the Laue condition is fulfilled for a lattice vector 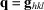.

Again, the detector captures the scattered intensity within the solid angle 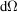, so that 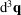 may be expressed following equation (6). For the Laue method, however, the reciprocal length element 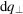 has to be expressed in terms of d*k*, as the detector intrinsically integrates over all the Ewald spheres related to the primary spectral intensity 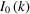. Using 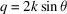 and 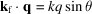, the reciprocal length element can be expressed as
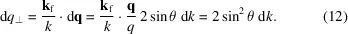
Here, it was assumed that d*k* only influences the length of the scattering vector 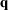, while its direction is not affected. Inserting equation (12) into equation (6), the widely recognized Lorentz factor for the Laue method is obtained as the Jacobian (Kalman, 1979 ▸; Lange, 1995 ▸):
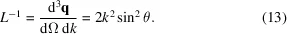
Here, the wavevector *k* is defined by the wavelength at which the Laue condition 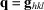 is fulfilled for a specific reflection *hkl*. This implies that, in contrast to the rotating single-crystal method, a Lorentz factor can only be applied to the measured data after successful peak indexing (*i.e.* the wavelength associated with the appearance of each reflection is required), emphasizing the complexity of data evaluation in a Laue diffraction experiment. A complementary derivation of the Lorentz factor for the Laue method is presented in Appendix *B*2[App appb], where the reciprocal-space vector is expressed as a function of the experimental variables and the Jacobian is calculated directly.

## 4. Powder X-ray diffraction

As the method of choice for the analysis of polycrystalline samples, powder X-ray diffraction is widely applied in both research and industry (Kaduk *et al.*, 2021 ▸). This is particularly related to the capabilities of the method for both structure elucidation and quantitative phase analysis, which both rely on accurately determined peak intensities.

In a polycrystalline sample, the diffracted intensity is influenced by the different contributing crystallites with various crystallite sizes and orientations. Correspondingly, for a polycrystalline sample equation (1) changes to
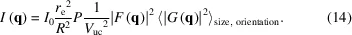
Here, 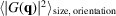 describes the geometrical factor, introduced in equation (3), averaged over the different crystallite sizes and orientations. For an ideal powder, the orientation distribution of crystallites may be considered isotropic, so that 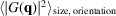 is constant for a defined length of the scattering vector 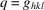. Correspondingly, the intensity distribution of equation (14) represents a set of concentric spheres with maximum intensity at the positions 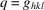, and their radial extensions determined by the size distribution of the involved crystallites.

Unlike in the case of the single crystal, where the integrated intensity of a reflection is obtained by integrating 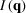 within a small volume of reciprocal space around the reflection, the integrated intensity of a reflection from a polycrystalline powder can only be obtained after integrating over the entire intensity sphere within a limited radial element 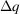. Correspondingly, for the integration, reciprocal spherical coordinates have to be used.

The structure factor 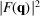 typically only depends on the length of the scattering vector, where 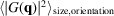 is sharply localized. Correspondingly 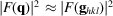 may be treated as constant and factored out of the integral. The remaining integration over the averaged geometrical factor yields
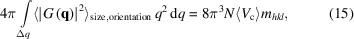
where *N* denotes the number of illuminated crystallites, 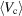 the average crystallite volume and 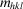 the multiplicity factor of the reflection. The prefactor 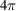 arises from the integration over the entire unit sphere. Combined with equation (14), the integrated intensity of a Bragg peak *hkl* from a polycrystalline powder is consequently defined by
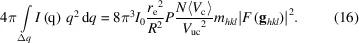


Correspondingly, the volume of irradiated crystallites 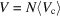 serves as a scattering invariant in powder X-ray diffraction. Any intensities obtained from a powder diffraction experiment have to be related to equation (16) using a respective Lorentz factor.

To derive the Lorentz factor for a powder diffraction experiment, we assume that the scattering angle 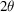 is varied by adjusting the angle of the incident and the scattered beam. Commonly, both angles are kept equally at half of the scattering angle, leading to a specular θ–2θ measurement geometry.

**Figure 2:**
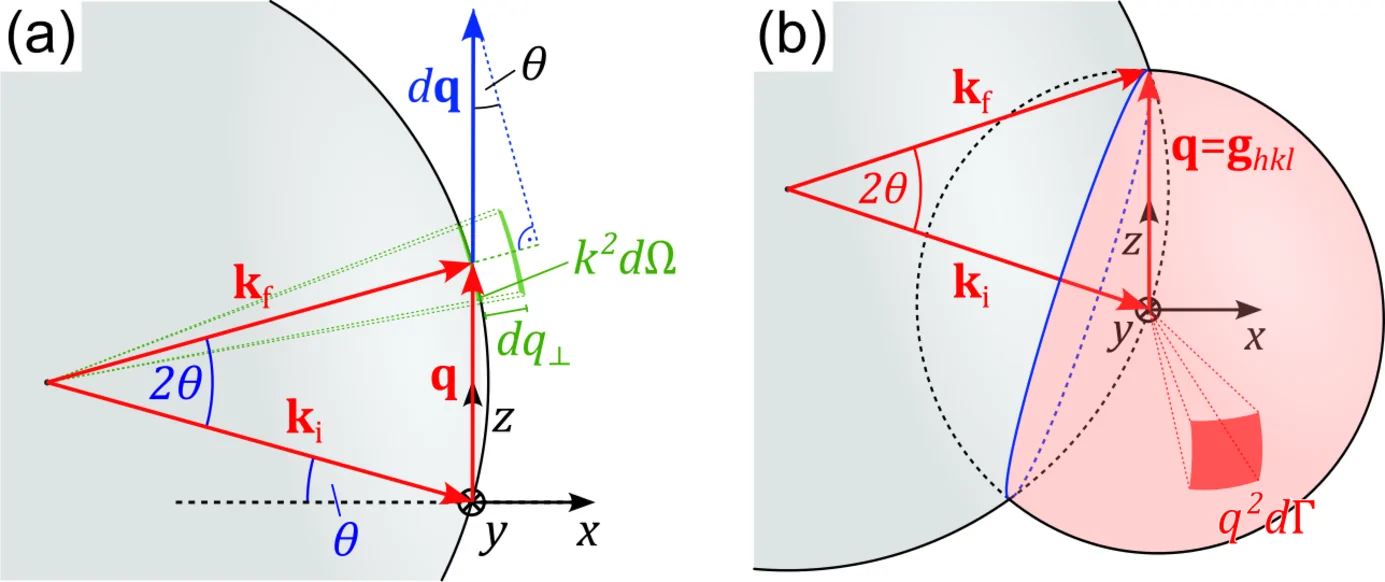
Reciprocal-space representation of a powder diffraction measurement for the derivation of the required Lorentz factor. (*a*) For a typical powder diffraction measurement, variation of the scattering angle 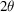 leads to a continuous change of the scattering vector 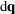. (*b*) The representation of randomly distributed crystallites in reciprocal space as reciprocal-lattice points assembled on the surface of a sphere (red) with radius 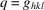. The intersection of this sphere with the Ewald sphere (gray) forms a Debye–Scherrer ring (blue), indicating that, at a specific scattering angle 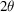, only a fraction of the illuminated crystallites in a powder are in the reflecting condition.

A reciprocal-space representation of such a measurement is shown in Fig. 2 ▸(*a*). The following derivation is based on a specular measurement geometry, although the obtained Lorentz factor is equally valid also for the non-specular case.

As a first step, a single crystal measured in the described measurement setup is considered, where equation (6) can be applied. As the scattering angle 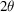 is varied as part of the experiment, the reciprocal length element 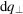 needs to be expressed in terms of a scattering angle element 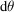. This is achieved using 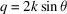 and 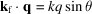, yielding
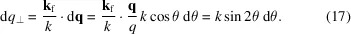


Consequently, the Lorentz factor for a single crystal measured in the powder diffraction setup is equal to the one obtained for the coplanar rotating single-crystal geometry, even though the scattering angle is varied during peak integration:
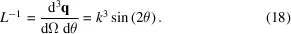


It is important to realize, however, that for a polycrystalline powder, the integration over the solid-angle element of the Ewald sphere 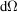 is not a suitable choice (Kalman, 1979 ▸). Instead, to obtain an integrated intensity that can be related to the scattering invariant 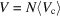, it is essential to determine the fraction of crystallites that contribute to scattering within the solid-angle element 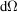 (Als-Nielsen & McMorrow, 2011 ▸). Mathematically, this corresponds to mapping the solid-angle element of the Ewald sphere 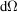 onto the solid-angle element 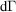 of the sphere of crystallite orientations.

Fig. 2 ▸(*b*) shows the Ewald sphere (gray) and the sphere of crystallite orientations (red). The intersection of the two spheres shown in blue represents the Debye–Scherrer ring for a specific set of planes 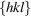. Along the Debye–Scherrer ring, the reciprocal area element 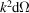 on the Ewald sphere can be projected onto the reciprocal area element 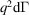 [see Fig. 2 ▸(*b*)] on the sphere of crystallite orientations. This projection yields a direct geometric relation between the respective solid-angle elements:
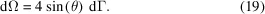
After inserting this relation into equation (18), the well known Lorentz factor of a polycrystalline powder is obtained as the Jacobian (Als-Nielsen & McMorrow, 2011 ▸; Lipson *et al.*, 2006 ▸):
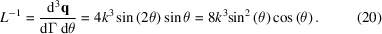
As the derived Lorentz factor has to be expressed in terms of the solid-angle element on the orientation sphere 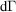, its validity is not limited to a specular θ–2θ scattering geometry, but it is equally applicable to powder patterns measured with fixed incident angle, or to static measurements on area detectors. Such measurements provide significant advantages over the conventional approach, as a powder can be measured in transmission or at a fixed angle of incidence and the entire diffraction pattern is obtained in a single shot. A conventional powder diffraction pattern given by measured intensity as a function of the scattering angle 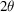 is then typically obtained via transformation of the measured data to reciprocal space and azimuthal integration using dedicated software tools (Ashiotis *et al.*, 2015 ▸).

The equivalence of the Lorentz factor for different scattering geometries can be directly observed upon close inspection of equation (16). Transforming the integral over the length of the scattering vector d*q* into an integral over the scattering angle 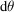, one immediately recognizes that 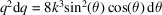. Consequently, the Lorentz factor of equation (20) can be derived directly from equation (16) without specific information about the scattering geometry.

Formally, the derived equation and the associated Lorentz factor are limited to ideal powders without texture. The influence of preferential orientation on the measured powder X-ray diffraction patterns may, however, be accounted for by introducing texture factors, for example based on spherical harmonics calculations (Von Dreele, 1997 ▸). To derive the specific Lorentz factors for highly textured polycrystalline samples, the case of a uniplanar (fiber) texture is discussed in Section 5.2, while more complex cases may be treated following the literature (Cella *et al.*, 1970 ▸; Kalman, 1979 ▸).

## 5. Grazing-incidence X-ray diffraction

Grazing-incidence X-ray diffraction is the state-of-the-art method to obtain crystallographic information from thin films and surfaces. This includes the study of the crystallization properties of various organic and inorganic compounds on surfaces, the connected appearance of substrate-induced polymorphs as well as the investigation of surface reconstructions (Werzer *et al.*, 2024 ▸). For any grazing-incidence measurement, obtaining reliable peak intensities comparable with calculated structure factors is essential, emphasizing the importance of accurate intensity correction factors.

**Figure 3:**
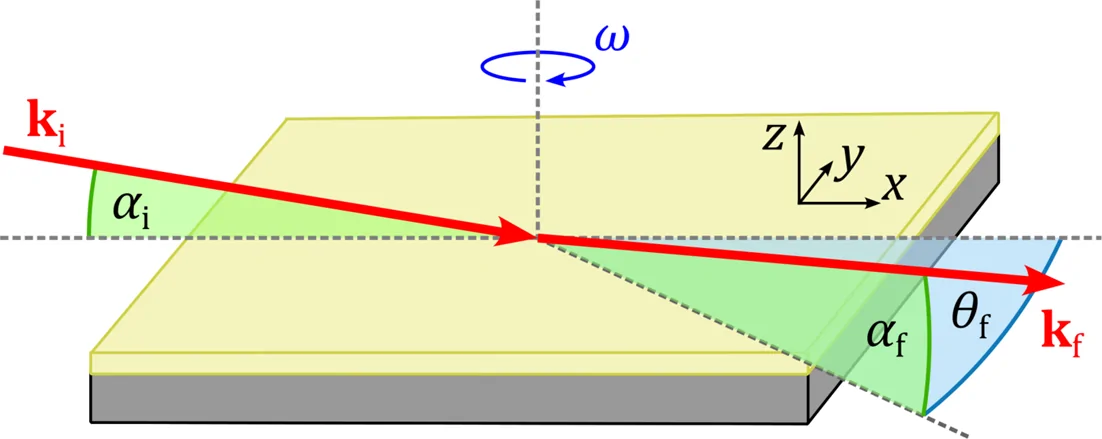
Scattering geometry of a grazing-incidence X-ray diffraction experiment. The incident wavevector 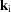 illuminates the sample at an incidence angle 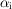, and the direction of the diffracted wavevector 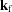 is defined by an in-plane angle 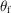 and an out-of-plane angle 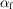. Typically, for surface X-ray diffraction measurements a sample rotation with angular velocity 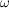 around the surface normal is performed.

Fig. 3 ▸ shows the general experimental setup of a grazing-incidence diffraction experiment. The X-ray beam illuminates the sample at a fixed incidence angle 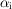, typically close to the critical angle of total external reflection. The diffracted X-rays are then detected for a defined angle 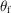 in the plane of the surface (*i.e.* the *xy* plane) and 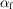 perpendicular to the thin film surface (*i.e.* the *z* direction). The incident and scattered wavevectors are, respectively, defined by
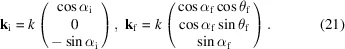


In the following, the Lorentz factor for two typical grazing-incidence diffraction measurements will be derived. For surface X-ray diffraction, the diffraction signal of a specific reflection is typically measured by rotating the sample about the surface normal over the entire width of a reflection. In contrast, grazing-incidence wide-angle X-ray scattering experiments frequently study polycrystalline thin films. Correspondingly, diffraction patterns can be recorded statically without the necessity of any sample rotation.

### 5.1. Surface X-ray diffraction

The diffraction signal of a single-crystalline surface can be expressed similar to equation (1),
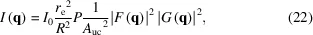
where 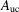 is the area of the surface unit cell. In contrast to the methods discussed so far, for surface diffraction only a limited number of repeating unit cells perpendicular to the substrate surface contribute to the diffracted intensity. As a consequence, the geometrical factor 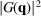 extends over a wide range in the *z* direction perpendicular to the surface, forming rod-like diffraction features (Robinson & Tweet, 1992 ▸). Correspondingly, the integration over the geometrical factor becomes
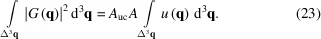
Here, *A* is the illuminated surface area and 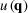 is a function that describes the intensity distribution along the 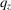 direction and is normalized such that its integral along 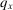 and 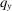 is unity. In many cases, 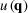 is not strictly determined by 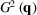, but is influenced by the correlation length on the surface, the mosaic spread, and other effects. The resulting integrated intensity of a diffraction rod is then given by
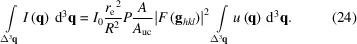


It is important to recognize that this result can only be obtained assuming that 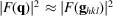 is approximately constant across the integration range 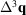. Along the in-plane directions 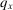 and 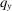 this is generally correct as the geometrical factor is limited in the substrate plane. Along 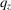, however, the integration range over the rod-like diffraction features needs to be limited experimentally. This approximation, and the remaining integration of 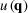 along 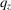, lead to an additional correction factor, denoted the rod interception factor, discussed in detail in the literature (Vlieg, 1997 ▸).

Nevertheless, it is still possible to derive a Lorentz factor if the integration of the geometrical factor in equation (23) is not performed in reciprocal-space coordinates. Conventionally, in a surface diffraction experiment, the intensity of a diffraction rod is measured with fixed detector position while rocking the sample over the entire reflection. This setup directly resembles the rotating single crystal, so that the Lorentz factor of a surface rocking scan can be derived directly following equation (8) (Vlieg, 1997 ▸; Smilgies, 2002 ▸):
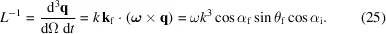
Hereby, it was assumed that the angular velocity 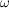 defines a rotation around the 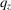 axis and the common definitions of the wavevectors 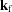 and the scattering vector 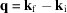 from equation (21) are used. The obtained Lorentz factor is valid for any rod scan other than the specular rod. For measurements of the specular rod, the in-plane angle is fixed to 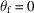 and the incident angle 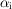 has to be varied, so that the Lorentz factor of equation (18) is applicable with 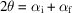.

### 5.2. Grazing-incidence wide-angle X-ray scattering

In grazing-incidence wide-angle X-ray scattering experiments, typically, polycrystalline samples are investigated. For an unoriented polycrystalline powder, the appropriate Lorentz factor has already been derived in equation (20) and remains equally applicable for measurements under grazing-incidence conditions.

In many cases, the presence of a substrate during thin film growth leads to a preferential orientation of the obtained crystallites in thin films. This may result in a uniplanar (fiber) texture (Heffelfinger & Burton, 1960 ▸; Roe & Krigbaum, 1964 ▸), in which crystallites have a defined orientation out of the substrate plane, while exhibiting isotropic orientation distribution within the substrate plane (Fischer *et al.*, 2023 ▸; Gasser, John *et al.*, 2025 ▸).

To describe the diffracted intensity distribution from a uniplanar-textured film, equation (14) can be used. In contrast to the unoriented powder, however, the averaged geometrical factor 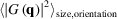 does not reflect concentric spheres in reciprocal space, but instead a set of concentric rings around the fiber axis. The center position of each ring is defined by 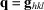, and its lateral width is determined by the crystallite size and the exact orientation distribution. Consequently, the integrated intensity of a given reflection must be obtained by integrating over the entire diffraction ring, for which reciprocal cylindrical coordinates are naturally useful. Similar to the powder case, 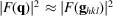 may be factored out of the integral, reducing the integration of equation (14) to an integral over the averaged geometrical factor:
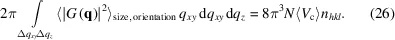
Here, *N* is the number of illuminated crystallites, 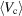 the average crystallite volume and 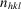 the multiplicity factor defined by the number of lattice planes contributing to the given diffraction ring. The in-plane component of the scattering vector is defined by 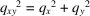, and its out-of-plane component 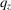 aligns with the fiber axis. The prefactor 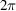 arises from the integration over the unit circle, with the azimuthal angle given by 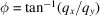.

The integrated intensity of a Bragg peak *hkl* from a polycrystalline uniplanar-textured thin film is therefore given by
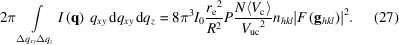


To derive the required Lorentz factor for a measurement performed in the angular coordinates introduced in equation (21), we determine 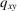 = 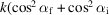

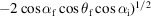
 and 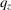 = 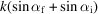. Consequently, the Lorentz factor is defined by the Jacobian:
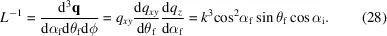
Here, we note that 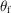 and 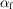 describe the azimuthal and polar angles of the Ewald sphere, respectively, so that a solid-angle element on the Ewald sphere is 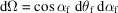. Correspondingly, the obtained Lorentz factor coincides with the one obtained in equation (25) for the surface rocking scan.

The obtained result of the Lorentz factor for a uniplanar-textured thin film strictly depends on the fiber axis and therefore cannot be generally applied to all polycrystalline thin films. In other words, for polycrystalline samples the Lorentz factor strictly depends on the specific texture of the sample additional to the measurement geometry. Accordingly, careful evaluation is required, and transforming measured data into reciprocal-space coordinates using software tools like *GIDVis* (Schrode *et al.*, 2019 ▸), *INSIGHT* (Reus *et al.*, 2024 ▸) or *PyFAI* (Ashiotis *et al.*, 2015 ▸) may be beneficial for a simplified and reliable data evaluation.

## 6. Small-angle X-ray scattering

Small-angle X-ray scattering (SAXS) is widely applied to investigate the organization of matter on the nanometre to the micrometre length scales (Guinier & Fournet, 1955 ▸; Feigin & Svergun, 1987 ▸; Jeffries *et al.*, 2021 ▸). In contrast to X-ray diffraction, the intensities measured in a SAXS experiment arise from spatial variations of the average electron densities inside a sample, leading to broad scattering features at small scattering vectors 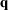.

In the framework of this work, two different classes of SAXS measurements can be distinguished. Firstly, SAXS is frequently used on poorly organized systems such as biological samples, solutions or gels. For such samples, no structural signal or Bragg peaks can be measured and equation (1) is not applicable. Instead, evaluation of SAXS data is only possible after careful investigation and modeling of the measured continuous small-angle intensity distribution.

Upon this evaluation, the integrated intensity of an entire SAXS pattern, denoted as the scattering invariant *Q*, is frequently determined (Kratky & Glatter, 1982 ▸; Gilmore *et al.*, 2019 ▸):
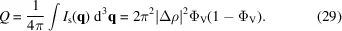
Here, 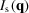 is the continuous intensity distribution of the SAXS signal in reciprocal space. The second part of the equation is only applicable to a two-level system, where 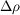 denotes the difference in the average electron density between the two components, and 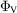 the respective volume fraction of the scattering features. For an isotropic sample, the intensity distribution 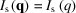 is isotropic, and the volume integral in equation (29) reduces to the one-dimensional integral over 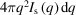, widely reported in the literature.

The importance of the scattering invariant *Q* is that it expresses a factor by which the data can be reduced to an absolute scale, which is essential for a successful interpretation of SAXS data. This idea was advanced for the study of weak scatterers, where an absolute intensity calibration procedure was proposed (Allen *et al.*, 2017 ▸). Hereby, the idea is to convert SAXS intensities into cm^−1^ in order to enable the comparison of SAXS data collected from various instruments, but mostly for subtraction of the signal of less interest (*e.g.* the signal of the solvent in solution scattering).

Similar to the definition of the integrated intensity in a diffraction experiment, the integrated SAXS intensity is defined as an integral in reciprocal space. Correspondingly, if a measured SAXS signal is integrated in any coordinate system other than reciprocal-space coordinates, a Jacobian accounting for the specific coordinate transformation is required. Conceptually, this Jacobian directly corresponds to the definition of the Lorentz factor for X-ray diffraction experiments, and some of the previously derived equations of the Lorentz factor may be equally applicable to specific SAXS measurements.

Secondly, SAXS can be used to measure highly periodic structures, leading to clear diffraction features at small scattering angles. Correspondingly, the method may be seen as an extension of X-ray diffraction to small angles, with similar peak integration approaches being applicable. As in SAXS the measured fraction of the Ewald sphere is small and therefore approximately flat, some of the previous descriptions can be significantly simplified (Cser, 2001 ▸).

For both types of experiments, it is important to realize, however, that the application of a Lorentz factor to measured SAXS data is strictly restricted to the calculation of an integral quantity like the scattering invariant *Q* or the integrated peak intensity in the case of small-angle diffraction. In contrast, applying a Lorentz factor to a continuous SAXS intensity distribution 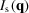 without integration is not justified, and can lead to incorrect data interpretation (Cser, 2001 ▸). Moreover, in the case of small-angle diffraction, any background scattering (*e.g.* air scattering) must be subtracted from the measured signal before a Lorentz correction can be applied and peak integration performed.

## 7. Discussion

The Lorentz factor arises when the integration over the intensity distribution 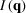 is carried out in any coordinate system other than reciprocal space. For the experimental techniques considered here, the corresponding real-space integration variables include the solid angle 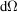 on the Ewald sphere defined by the detector element, the measurement time d*t*, specific angular measurement variables 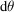, 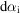, 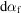, 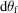, the length of the wavevector d*k*, and the angular variables 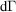, 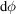 describing the orientation distribution of polycrystalline samples.

These contributions may be generalized into eight possible integration coordinates: the angular divergence of the primary beam 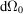, the spectral width of the primary beam d*k*, the solid angular opening of the detector 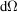, the measurement time d*t* and the orientation distribution of the crystallites in the sample 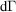. Correspondingly, the integrated intensity of a reflection in real space may be written as
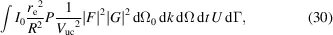
where 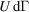 describes the probability that a reciprocal-lattice vector 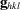 lies inside the elementary solid angle 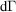. The selection of three appropriate integration variables for the derivation of the Lorentz factor strictly depends on the experimental geometry and the nature of the sample, and can be described by three fundamental criteria (Kalman, 1979 ▸):

(i) The integration variables must result in a finite, non-vanishing Jacobian.

(ii) The integration variables must vary sufficiently during the measurement, so that their integration range covers the whole extent of a reflection given by the geometrical factor 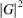.

(iii) The structure factor 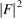, the orientation distribution function *U* (for polycrystalline samples), as well as the primary spectral intensity 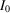 (when using polychromatic radiation), must not depend significantly on the integration variables within their integration range.

The resulting expressions of the Lorentz factor for different X-ray diffraction techniques generally share a common structure. Specifically, the Lorentz factor can be separated into a trigonometric component, which depends on the angular coordinates of the measurement, and a wavelength-dependent component. For measurements performed with strictly monochromatic radiation at a fixed wavelength, the wavelength-dependent contribution reduces to a constant factor and can therefore be omitted when relating relative integrated intensities to calculated structure factors. In contrast, the wavelength-dependent term becomes particularly important when comparing data acquired at different X-ray energies, as in resonant or anomalous diffraction experiments (Collins & Gann, 2022 ▸; Attfield, 1996 ▸; Cianci *et al.*, 2005 ▸; Stragier *et al.*, 1992 ▸).

In the literature, commonly only the trigonometric contribution is identified as the Lorentz factor, whereas the wavelength dependence is treated separately. It is important to recognize, however, that both contributions arise from the same underlying principle, namely that the measured integrated peak intensities need to be related to the wavelength- and angle-independent reciprocal-space coordinates.

The availability of fast numerical processing methods makes it possible to transform experimental diffraction data directly into reciprocal space, enabling peak integration according to equation (5) without application of a Lorentz factor. However, such transformations typically require substantially increased computational effort and are therefore often impractical for routine data evaluation. In addition, diffraction experiments are frequently performed in a continuous scanning mode, in which the detector intrinsically integrates the diffracted intensity over a small angular range. This includes, for example, single-crystal and surface diffraction experiments with continuous crystal rotation, as well as continuous θ–2θ scans of polycrystalline powders. For data acquired in this manner, peak integration must be performed in the measurement coordinates, requiring the application of an appropriate Lorentz factor.

In contrast, for measurements performed in step-by-step scanning mode, peak integration in reciprocal space can, in certain cases, be advantageous. This is particularly the case for static measurements of powders or uniplanar-textured thin films. Here, only a single detector frame needs to be transformed to reciprocal space, so that data evaluation in reciprocal spherical or cylindrical coordinates can be performed without significant increase in computation time (Gasser, Simbrunner *et al.*, 2025 ▸).

Nevertheless, this approach is only applicable if the polycrystalline samples can be described by an idealized orientation distribution like an ideal powder or an ideal uniplanar texture. In the general case of a complex orientation distribution of the crystallites, a complete integrated intensity of a reflection can only be obtained after determination of the respective orientation distribution using complementary tools, such as pole figure measurements (Schulz, 1949 ▸; Mocuta *et al.*, 2013 ▸) or by detailed computational texture analysis (Von Dreele, 1997 ▸).

## 8. Conclusion

In this review, the origin, derivation and application of the Lorentz factor for X-ray diffraction experiments have been systematically examined across a broad range of experimental geometries and sample types. Starting from the fundamental definition of integrated intensities in reciprocal space, it was demonstrated that the Lorentz factor naturally emerges as the Jacobian associated with transforming the experimental (real-space) integration variables into reciprocal-space coordinates. Within this framework, the widely used interpretation of the Lorentz factor as an ‘angular-velocity factor’ follows directly when the sample or the detector are rotated as part of the diffraction experiment.

The presented perspective provides a unified framework for understanding the Lorentz factor across conventional diffraction methods, grazing-incidence techniques, as well as small-angle scattering experiments. The examples of the Lorentz factor for the rotating single crystal, the Laue method, powder diffraction and grazing-incidence diffraction demonstrate that the Lorentz factor is not a universal quantity, but depends sensitively on the experimental geometry as well as the type of sample under investigation (*i.e.* single crystals, powders or textured materials). In this context, the Lorentz factor was shown to be equally relevant for experiments involving sample or detector rotations and static measurement geometries. Furthermore, it was shown that the same concept is also applicable to SAXS, where the Lorentz factor can be useful for calculation of the integrated SAXS intensity.

This review emphasizes that, despite the wide availability of advanced computational tools for data processing, real-space peak integration combined with an appropriate Lorentz factor remains indispensable for many experimental X-ray diffraction investigations, particularly for continuous scanning geometries. At the same time, direct integration in reciprocal space, without the need for a Lorentz factor, can provide a valuable alternative for data evaluation.

## Appendix A

### A1. Derivation of the integral over the geometrical factor |*G*(**q**)|^2^

Following Parseval’s theorem, the integral of the squared modulus of the Fourier transform of a function over reciprocal space equals  times the integral of the squared modulus of the original function over real space. Correspondingly, the integration over the geometrical factor  in reciprocal space can be reduced to an integration over the crystal shape function  in real space:

Here, the summation over the Laue indices *hkl* is dropped as the integration limits  are defined to contain only a single reciprocal-lattice point. Finally, the crystal shape function is defined as unity inside, and zero outside the crystal, so that , and its integration over real space simply yields the volume of the crystal .

### A2. Alternative intensity formulation for overlapping reflections

For small crystals, neighboring reflections will significantly overlap, making it impossible to define a finite reciprocal-space volume  required for the calculation of integrated intensities following equations (3) and (4). An alternative formulation can be achieved by defining the intensity distribution of a single reflection,

with  denoting the intensity of the incident X-ray beam,  the classical electron radius, *R* the distance from the crystal to the detector, *P* the polarization factor,  the unit-cell volume, *F* the structure factor and *S* the crystal shape function. Similar to equation (5), the integrated intensity of a reflection can now be directly related to the crystallite volume  as follows:

## Appendix B

### b1. Algebraic derivation of the Lorentz factor for the rotating single crystal

An alternative algebraic derivation of the Lorentz factor for the rotating single-crystal method can be achieved by defining the incident and scattered wavevectors, respectively, as

where  is the scattering angle between  and , and  is the azimuthal angle around . To include the rotation of the crystal in the description, the scattering vector  can be expressed in the crystal reference frame. This is achieved by multiplying the scattering vector  by a rotation matrix,

describing a rotation of the crystal around the *y* axis, as visualized in Fig. 1 ▸(*a*), with an angular velocity . As a result, the scattering vector in the crystal reference frame is given by

Based on this expression, the Jacobian for the transformation from the reciprocal-space coordinates  to the real-space coordinates  can be calculated directly without any further geometrical considerations. Correspondingly, for the rotating single-crystal method the Lorentz factor

is obtained. Here, we note that the solid-angle element on the Ewald sphere is , so that

Importantly, the exact same result is achieved by inserting the expressions for  and  from equation (34) into equation (8), showing the direct equivalence between both approaches. For , the setup corresponds to a coplanar scattering geometry, and equation (38) gives the simple  dependence, in analogy to equation (10).

### b2. Algebraic derivation of the Lorentz factor for the Laue method

Following equation (34), the scattering vector can be expressed in the laboratory reference frame as

Based on this expression, the Lorentz factor can be calculated directly as the Jacobian for a transformation from the reciprocal-space coordinates  to the real-space coordinates . Correspondingly, the Lorentz factor

is obtained, where we note that the solid-angle element on the Ewald sphere is , so that the obtained expression is equivalent to equation (13).
